# HIV-1 Vpr Abrogates the Effect of TSG101 Overexpression to Support Virus Release

**DOI:** 10.1371/journal.pone.0163100

**Published:** 2016-09-20

**Authors:** Nopporn Chutiwitoonchai, Lowela Siarot, Eri Takeda, Tatsuo Shioda, Motoki Ueda, Yoko Aida

**Affiliations:** 1 Viral Infectious Diseases Unit, RIKEN, Wako, Saitama, Japan; 2 Japan Foundation for AIDS Prevention, Chiyoda-ku, Tokyo, Japan; 3 Graduate School of Agricultural and Life Sciences, The University of Tokyo, Bunkyo, Tokyo, Japan; 4 Research Institute for Microbial Diseases, Osaka University, Suita, Osaka, Japan; 5 Nano Medical Engineering Laboratory, RIKEN, Wako, Saitama, Japan; University of Alabama at Birmingham, UNITED STATES

## Abstract

HIV-1 budding requires interaction between Gag and cellular TSG101 to initiate viral particle assembly and release via the endosomal sorting complexes required for transport (ESCRT) pathway. However, some reports show that overexpression of TSG101 inhibits virus release by disruption of Gag targeting process. Since a HIV-1 accessory protein, Vpr binds to Gag p6 domain at the position close to the binding site for TSG101, whether Vpr implicates TSG101 overexpression effect has not been investigated. Here, we found that Vpr abrogates TSG101 overexpression effect to rescue viral production. Co-transfection of TSG101 and Gag with Vpr prevented TSG101-induced Gag accumulation in endosomes and lysosomes. In addition, Vpr rescued virus-like particle (VLP) production in a similar manner as a lysosomal inhibitor, Bafilomycin A1 indicating that Vpr inhibits TSG101-induced Gag downregulation via lysosomal pathway. Vpr and Gag interaction is required to counteract TSG101 overexpression effect since Vpr A30F mutant which is unable to interact with Gag and incorporate into virions, reduced ability to prevent Gag accumulation and to rescue VLP production. In addition, GST pull-down assays and Biacore analysis revealed that Vpr competed with TSG101 for Gag binding. These results indicate that Vpr overcomes the effects of TSG101 overexpression to support viral production by competing with TSG101 to bind Gag.

## Introduction

Human immunodeficiency virus type 1 (HIV-1) Gag protein is initially translated as a p55 Gag precursor (Pr55 Gag), which is then cleaved by viral protease into the p17 matrix (MA), the p24 capsid (CA), the p7 nucleocapsid (NC), and p6 proteins during viral maturation. MA, CA, and NC are structural proteins. The MA domain is N-terminal myristoylated to traffic Pr55 Gag to the plasma membrane, while the NC domain recruits viral genomic RNA [1, 2]. The p6 domain plays two important roles: viral assembly/budding and incorporation of Vpr into virions. HIV-1 assembly/budding occurs via the cellular endosomal sorting complexes required for transport (ESCRT) machinery [3–5]. The ESCRT machinery is a multi-protein complex comprising ESCRT-0, ESCRT-I, ESCRT-II, ESCRT-III, and ESCRT accessory subunits; this machinery functions during biogenesis of multivesicular bodies (MVBs), cytokinesis, and macroautophagy [6, 7]. HIV-1-hijacked ESCRT has been described in many reports [8–13]. The process is initiated via interaction between the late (L)-domain (the PTAP motif located at the Gag p6 domain) and the ubiquitin E2 variant (UEV) domain of Tumor Susceptibility Gene 101 (TSG101), a component of ESCRT-I. The Gag p6 domain then recruits AIP1 (or ALIX; an ESCRT accessory protein) via its alternative L-domain, LYPX_n_L [14, 15]. These interactions result in recruitment of ESCRT-III proteins, i.e., charged MVB protein 1 (CHMP1), CHMP2, and CHMP4, to the assembly site to form the viral particle budding neck at the plasma membrane. The final step of virus budding is mediated by recruitment of vacuolar protein sorting-associated protein 4 (VPS4) ATPase, which is required for the membrane fission step that allows virion release from the plasma membrane.

Some reports have identified another role for the Gag p6 domain: the incorporation of Vpr, an accessory HIV-1 protein, into the virion via its ^15^FRFG^18^, ^34^ELY^36^, and ^41^LXSLFG^46^ domains [16–19]. Virion-incorporated Vpr promotes viral infectivity, replication, and AIDS disease progression [20–22]. Vpr also has other critical functions, including induction of G_2_ cell cycle arrest [23, 24], modulation of apoptosis [25], activation of HIV-1 long terminal repeat (LTR) transcription [26], and regulation of cellular pre-mRNA splicing [27].

Although TSG101 plays a role in the HIV-1 assembly/budding process, overexpression of full-length TSG101 inhibits HIV-1 release [28, 29]. In addition, overexpression of the N-terminal sequence of TSG101 in a dominant-negative manner blocks the function of viral Gag PTAP, whereas the C-terminal TSG101 sequence disrupts the cellular endosomal sorting pathway [28–30]. Since the Gag p6 domain contains both binding sites for TSG101 and Vpr, whether Gag/Vpr interaction affects Gag/TSG101 binding has not been elucidated. In addition, the correlation between these two interactions may reveal additional information that increases our understanding of the HIV-1 assembly process.

Here, we performed a detailed analysis of Vpr effect on TSG101 overexpression-mediated defective in viral release and found that Vpr competes with TSG101 for Gag binding to prevent Gag accumulation and Gag degradation in endosomal/lysosomal pathway thereby rescuing virion release.

## Materials and Methods

### Cell culture and transfection

HEK293T and HeLa cells were maintained in Dulbecco’s Modified Eagle’s Medium (Gibco Life Technologies) supplemented with 10% fetal bovine serum (Gibco Life Technologies; and HyClone Laboratories) under 5% CO_2_/37°C conditions. FuGENE HD (Promega) and Lipofectamine 3000 (Invitrogen) were used for plasmid transfections throughout the study.

### Plasmid construction

Construction of all plasmids is described in S1 File.

### Immunofluorescence staining

HeLa cells (4 × 10^4^) were seeded overnight on cover glass in 12-well plates before transfection for 48 h with 0.8 μg of pCAGGS/Gag and 1.5 μg of pCAGGS/eCFP-TSG101 without/with 0.5 μg of pCAGGS/HA-Vpr, 0.5 μg of pCAGGS/HA-Vpr A30F, or 0.5 μg of pCAGGS/Vif-HA plasmids. After 48 h transfection, the cells were processed to immunofluorescent stain as described previously [31] with the following antibodies: anti-Gag rabbit polyclonal antibody (pAb) (Bio Academia), anti-HA mouse monoclonal antibody (mAb) (Medical and Biological Laboratories Co., LTD), anti-LAMP1 mouse mAb (Santa Cruz Biotechnology), anti-EEA1 rabbit mAb (Cell Signaling), anti-Rab7 rabbit mAb (Cell Signaling), anti-Rab11 rabbit pAb (Invitrogen), Alexa Fluor 594 goat anti-rabbit IgG (Invitrogen), and Alexa Fluor 633 goat anti-mouse IgG (Invitrogen). Image acquisition was performed at the followed excitation/emission wavelengths (Ex./Em.): Alexa Fluor 594 at 543/618 nm, Alexa Fluor 633 at 635/647 nm, and eCFP at 405/476 nm, under a confocal laser-scanning microscope (IX81-FV1000-D/FLUOVIEW System, Olympus). Gag/TSG101 co-localization was analyzed by Pearson’s correlation coefficients (linear regression of Gag and TSG101 fluorescent intensity plot which 1 is a total positive correlation, 0 is no correlation, and −1 is a negative correlation) using FV10-ASW v.2.1 software (Olympus).

### Fluorescence resonance energy transfer (FRET) analysis

HeLa cells (4 × 10^4^) were transfected with 0.8 μg of pCAGGS/Gag-Venus (or pCAGGS/Venus only) or with 1.5 μg of pCAGGS/eCFP-TSG101 (or pCAGGS/eCFP only) without/with 0.5 μg of pCAGGS/mRFP-Vpr plasmids for 48 h, fixed with 4% paraformaldehyde, and then visualized using a IX81-FV1000-D/FLUOVIEW System (Olympus). The fluorescent signal generated by the eCFP donor was acquired at an Ex. of 405 nm (Ex.eCFP) and an Em. of 460–500 nm (Em.eCFP). The fluorescent signal generated by the Venus acceptor was acquired at Ex. of 515 nm (Ex.Venus) and Em. of 515–615 nm (Em.Venus). The uncorrected FRET signal was acquired at the Ex.eCFP and Em.Venus. The precision FRET (PFRET) signal was generated using the sensitized emission method by subtracting the uncorrected FRET signal from the signal generated by spectral bleed-through into the acceptor channel (the fluorescent signal from the donor emitted into the acceptor channel and the fluorescent signal generated by excitation of the acceptor at the donor excitation wavelength), which is derived from eCFP-TSG101 only and Gag-Venus only samples [32]. This process was automatically calculated by FV10-ASW v.2.1 software (Olympus). The FRET ratio was calculated by dividing the PFRET signal with the donor eCFP-TSG101 signal.

### Total internal reflection fluorescence (TIRF) microscopy

HeLa cells (5 × 10^4^) were seeded on cover glass in 24-well plates and transfected with 0.3 μg of pNL43 Luc E^-^ R^+^ or pNL43 Luc E^-^ R^-^, either without/with 0.1 μg of pCAGGS/mRFP-TSG101 and 0.1 μg of pcDNA/YFP-Vpr plasmids, for 16 h. The cells were then fixed in 3.7% formaldehyde, permeabilized with 0.5% Triton X100, and stained with anti-p24 mouse mAb AG3.0 [33] and FITC-conjugated sheep anti-mouse Fab’ (ICN Biomedicals). TIRF images were acquired by an Eclipse TE2000-E inverted microscope (Nikon).

### Analysis of virus-like particle (VLP) and viral particle production

VLPs were produced by transfection of HEK293T cells (8.3 × 10^5^ in 6-well plates) with 0.8 μg of pCAGGS/Gag and 1.5 μg of pCAGGS/FLAG-TSG101, either without/with the indicated amount of pCAGGS/Vpr or pCAGGS/Vpr A30F plasmids for 48 h (see the Figures). VLPs in culture medium were purified by 20% sucrose cushion method [34]. Briefly, the culture medium was collected and underlayered with 20% sucrose prior to ultracentrifugation at 40,000 rpm for 30 min at 4°C. After removing the supernatant, NET lysis buffer (10 mM Tris-Cl pH 7.4, 150 mM NaCl, 1 mM EDTA, 1% NP-40) and 4 × SDS sample buffer were added to the VLP pellet for resuspension prior to boiling and western blot analysis. The transfected cells were collected, washed with phosphate buffered saline (PBS), lysed with NET buffer (supplied with a complete protease inhibitor cocktail; Roche) on ice for 30 min, clarified by centrifugation at 15,000 rpm, and boiled with 4 ×SDS sample buffer.

Viral particles were produced by transfecting HEK293T cells with 0.2 μg of pNL43 Luc E^-^ R^+^ or pNL43 Luc E^-^ R^-^ and 1 μg of pCAGGS/FLAG-TSG101, either without/with the indicated amount of pCAGGS/Vpr plasmids, for 48 h. Culture medium containing viral particles was collected, mixed with 1/5 volume of PEG-it virus precipitation solution (System Biosciences), and stored at 4°C overnight before centrifugation at 1,500 × g for 30 min at 4°C. The supernatant was then removed, and the virus pellet was resuspended in NET lysis buffer and 4 × SDS sample buffer before boiling for western blot analysis. Whole cell lysates of producer cells were prepared as described above.

For the experiment involving Bafilomycin A1 (BFA1) (Sigma-Aldrich) and Clasto-lactacystin β-lactone (LC) (Cayman Chemical) inhibitors, cells were transfected with the indicated plasmids for 24 h before addition of the inhibitor and cultured for a further 6 h.

### GST pull-down assay

GST and GST-Gag were prepared by transformation B21 competent *E*. *coli* with pGEX-6P-3 or pGEX-6P-3/Gag plasmid and overnight culture, followed by inducing the bacterial culture (at the optical density of 600 nm = 0.1) with 1 mM Isopropyl β-D-1-thiogalactopyranoside for 3 h. Bacterial cells were harvested, lysed with BugBuster Protein Extraction Reagent (Novagen) at room temperature for 20 min, and the GST protein was immobilized by incubating the lysate with Glutathione Sepharose 4 Fast Flow beads (GE Healthcare Bio-Sciences AB) at room temperature for 1 h. The beads were then washed with PBS and resuspended with PBS to yield a 50% slurry.

FLAG-TSG101 and HA-Vpr proteins were prepared from HEK293T cells transfected with pCAGGS/FLAG-TSG101 or pCAGGS/HA-Vpr plasmid for 48 h. The cells were collected, lysed with NET buffer (supplied with a protease inhibitor), and incubated with anti-FLAG M2 affinity gel (Sigma-Aldrich; for FLAG-TSG101) or anti-HA agarose (Sigma-Aldrich; for HA-Vpr) at 4°C for 3 h. Next, HA-Vpr or FLAG-TSG101 protein was eluted from the agarose beads by co-incubation with 100 μg/ml of FLAG peptide (Sigma-Aldrich; for FLAG-TSG101) or HA peptide (Sigma-Aldrich; for HA-Vpr) at 4°C for 3 h.

A GST pull-down assay was performed by co-incubating 10 μl of GST or GST-Gag beads with 1 μg of FLAG-TSG101 protein either without/with PTAP short peptide (amino acid sequence; PEPTAPPEE, Scrum) [35], BSA, or HA-Vpr protein in 500 μl of EBC buffer (50 mM Tris-Cl pH 8.0, 140 mM NaCl, 0.5% NP-40, supplied with a protease inhibitor) overnight at 4°C. The beads were then pulled-down, washed with EBC buffer, and boiled with 4 × SDS sample buffer prior to western blot analysis. Pre-incubation was performed by incubating GST-Gag beads with PTAP short peptide or HA-Vpr protein for 3 h at 4°C before addition of FLAG-TSG101 protein and overnight incubation.

### Western blot analysis

Protein samples were separated by SDS-polyacrylamide gel electrophoresis and transferred onto PVDF membrane as described elsewhere [31]. The following antibodies were used: anti-Gag rabbit pAb (Bio Academia), anti-Vpr mouse mAb (Cosmo Bio Co., LTD), anti-FLAG M2 mouse mAb (Sigma-Aldrich), anti-GST rabbit pAb (Medical and Biological Laboratories Co., LTD), anti-GST goat pAb (GE Healthcare), anti-β-actin mouse mAb (Sigma-Aldrich), horseradish-peroxidase (HRP)-conjugated goat anti-mouse IgG (Amersham Biosciences), HRP-conjugated goat anti-rabbit IgG (Amersham Biosciences), and HRP-conjugated donkey anti-goat IgG (Santa Cruz Biotechnology). The intensity of the protein bands was measured using an ImageJ v.1.50 software.

### Biacore analysis

GST-Gag protein was prepared as described above and immobilized (100 μg/ml injection) on Biacore sensor chip CM5 (GE Healthcare) at 6,000–7,000 response units (RU) using Amine coupling kit (GE Healthcare) and 10 mM sodium acetate, pH 4.5 by Biacore T100 instrument (GE Healthcare). A reference surface was also activated and deactivated by amine coupling reaction without protein addition. FLAG-TSG101 and FLAG-Vpr proteins were prepared as described previously [31] and purified with PD-10 desalting column (GE Healthcare) followed by 10K molecular weight cut-offs Amicon Ultra centrifugal filter (Milipore). A serial dilution of FLAG-TSG101 or FLAG-Vpr protein at 0, 15.6, 31.3, 65.5, 125.0, 250.0, and 500.0 nM in 1 × HBS-EP+ buffer (GE Healthcare) were injected over the GST-Gag immobilized and reference surfaces at contact time = 180 s, flow rate = 10 μl/min, 25°C. The equilibrium dissociation constant (KD) of Gag/TSG101 and Gag/Vpr binding kinetic and affinity were analyzed by Biacore T100 Evaluation v.2.0.4 software (GE Healthcare) using the fit models: 1:1 Binding kinetic and Steady Stage Affinity. An unreliable point was excluded before the analysis.

## Results

### Vpr abrogates TSG101 overexpression effects to rescue VLP production via prevention of Gag accumulation and degradation in lysosome

**Fig 1:**
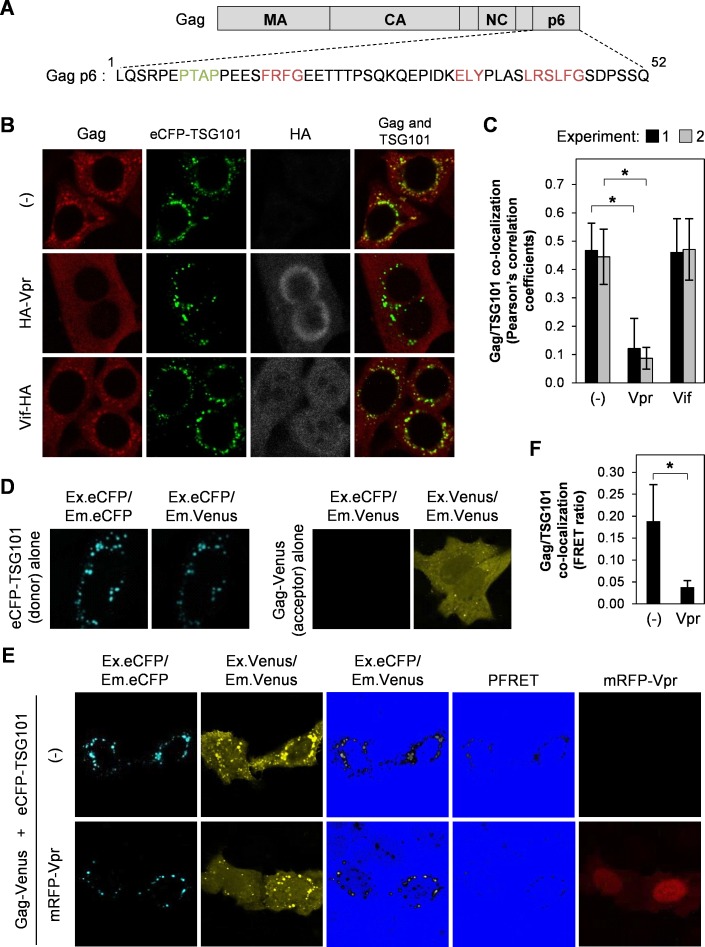
Vpr prevents TSG101-induced Gag accumulation at perinuclear region. (A) Schematic showing the binding motifs for TSG101 (PTAP; green) and Vpr (FRFG, ELY, and LXSLFG; red) within the Gag p6 domain. (B) HeLa cells were co-transfected with 0.8 μg of pCAGGS/Gag and 1.5 μg of pCAGGS/eCFP-TSG101 either without/with 0.5 μg of pCAGGS/HA-Vpr or 0.5 μg of pCAGGS/Vif-HA for 48 h prior to immunofluorescence staining with an anti-Gag and anti-HA antibodies, followed by an Alexa Fluor 594 goat anti-rabbit antibody and an Alexa Fluor 633 goat anti-mouse antibody. Image acquisition was performed under a confocal laser-scanning microscope. (C) Co-localization of Gag/TSG101 was analyzed by Pearson’s correlation coefficients. Data represent the means ± SD of the result of two independent experiments. *, P < 0.05 (unpaired t-test). (D-E) HeLa cells were co-transfected with 1.5 μg of pCAGGS/eCFP-TSG101 or 0.8 μg of pCAGGS/Gag-Venus (D) or 1.5 μg of pCAGGS/eCFP-TSG101 and 0.8 μg of pCAGGS/Gag-Venus either without/with 0.5 μg of pCAGGS/mRFP-Vpr (E) for 48 h before fixation and image acquisition. The precision FRET (PFRET) signal was analyzed using the sensitized emission method. The FRET (Ex.eCFP/Em.Venus) and PFRET image colors were converted by Hi/Lo function of FV10-ASW v.2.1 software (Olympus) to facilitate visualization of Gag/TSG101 co-localization. (F) FRET ratio (PFRET signal divided by the donor eCFP-TSG101 signal) of Gag/TSG101 co-localization in the absence or presence of Vpr. Data represent means ± SD of the result of one representative experiment from two independent performs. *, P < 0.05 (unpaired t-test).

TSG101 plays a role in HIV-1 assembly and budding by interacting with the PTAP L-domain of Gag p6. However, overexpression of full-length TSG101 causes defective virus release via disruption of endosomal sorting machinery [29]. In addition, full-length TSG101 overexpression decreases accumulation of Gag precursor in plasma-membrane-enriched fraction while increases cleavage-intermediated and matured Gag in early-endosome-enriched fraction [28]. The Gag p6 domain is also required for Vpr interaction to incorporate Vpr into viral particles [16–19] and Fig 1A shows the close binding site of Vpr (^15^FRFG^18^) and TSG101 (^7^PTAP^10^) on Gag p6 domain. Therefore, we speculated that Vpr/Gag interaction may interfere with TSG101/Gag binding, thereby inhibiting TSG101 overexpression effect. We first examined co-localization of TSG101 and Gag in Vpr-transfected HeLa cells and found that Vpr prevented TSG101-induced Gag co-accumulation by analysis of Pearson’s correlation coefficients (Fig 1B and 1C). This inhibition effect did not occur in the presence of Vif (as a negative control) which does not interact with Gag (Fig 1B and 1C). In addition, co-transfection of Gag PTAP domain mutant (PTAP to LIRL, which do not interact with TSG101) and TSG101 did not result in Gag accumulation (S1A Fig). These indicate the involvement of Vpr inhibitory effect and Gag/TSG101 interaction. We next verified the inhibitory effect of Vpr by FRET analysis using pCAGGS/Gag-Venus, pCAGGS/eCFP-TSG101, and pCAGGS/mRFP-Vpr plasmids. Transfection of pCAGGS/eCFP-TSG101 (donor) alone or pCAGGS/Gag-Venus (acceptor) alone, followed by image acquisition of eCFP, Venus, and FRET signals, was used as the control for PFRET analysis by the sensitized emission method [32] (Fig 1D). The PFRET signals confirmed co-localization of Gag/TSG101 and this co-localization was inhibited by Vpr (Fig 1E). In addition, comparison of the FRET ratio (the PFRET signal divided by the eCFP signal generated by the donor eCFP-TSG101) confirmed that the co-localization of Gag and TSG101 was reduced in the presence of Vpr (Fig 1F). The mRFP only plasmid (pCAGGS/mRFP) as a negative control did not inhibit Gag/TSG101 co-accumulation (S1B Fig). These results indicate that Vpr prevents Gag accumulation affected by TSG101 overexpression.

**Fig 2:**
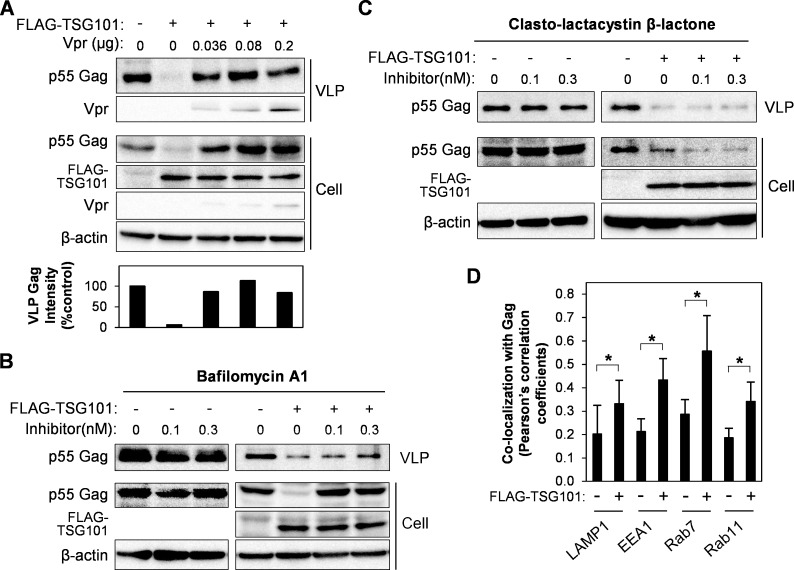
Vpr rescues VLP production by inhibition of TSG101-induced Gag degradation via lysosomal pathway. (A) HEK293T cells were transfected with 0.8 μg of pCAGGS/Gag, or with 0.8 μg of pCAGGS/Gag plus 1.5 μg of pCAGGS/FLAG-TSG101 either without/with different amounts of pCAGGS/Vpr for 48 h. VLPs in the cultured medium were collected by a 20% sucrose cushion. Whole cell lysates were prepared and samples were subjected to western blot analysis with anti-Gag, anti-Vpr, anti-FLAG, and anti-β-actin antibodies. The bottom panel represents intensity of VLP Gag from western blot analysis. Data represent the result of one representative experiment from three independent performs. (B-C) HEK293T cells were transfected with 0.8 μg of pCAGGS/Gag, or with 0.8 μg of pCAGGS/Gag and 1.5 μg of pCAGGS/FLAG-TSG101, for 24 h, before addition of Bafilomycin A1 (B) or Clasto-lactacystin β-lactone (C), followed by further culture for 16 h. VLPs and whole cell lysates were prepared and subjected to western blot analysis with anti-Gag, anti-Vpr, anti-FLAG, and anti-β-actin antibodies. Data represent the result of one representative experiment from two independent performs. (D) HeLa cells were transfected with 0.8 μg of pCAGGS/Gag-Venus, or with 0.8 μg of pCAGGS/Gag-Venus plus 1.5 μg of pCAGGS/eCFP-TSG101, for 48 h, followed by immunofluorescence staining anti-LAMP1, anti-EEA1, anti-Rab7, or anti-Rab11 antibody with a secondary antibody, Alexa Fluor 594 goat anti-rabbit antibody. Co-localization of Gag/LAMP1, Gag/EEA1, Gag/Rab7, or Gag/Rab11 was analyzed by Pearson’s correlation coefficients. Data represent means ± SD of the result of one representative experiment. *, P < 0.05 (unpaired t-test).

We further expected that, if Vpr inhibits Gag/TSG101 co-accumulation, then it should rescue VLP production in TSG101-transfected cells. Co-transfection of HEK293T cells with pCAGGS/Vpr along with pCAGGS/FLAG-TSG101 and pCAGGS/Gag rescued VLP production in the culture medium (Fig 2A). In addition, Vpr also prevented downregulation of intracellular Gag from TSG101 overexpression effect. This Gag downregulation was also previously observed when using high TSG101 plasmid amount [30]. To examine the mechanism by which TSG101 downregulates Gag, we treated cells with two inhibitors of cellular protein degradation pathways, BFA1 (a lysosomal degradation inhibitor) [36] or LC (a proteasomal degradation inhibitor) [37]. The inhibitors were added to HEK293T cells 24 h after transfection with pCAGGS/Gag and pCAGGS/TSG101 plasmids, followed by culture for a further 6 h. Western blotting showed that BFA1 rescued Gag downregulation and VLP production in TSG101-transfected cells while LC did not (Fig 2B and 2C). No effect of BFA1 or LC on Gag expression in the Gag only transfected cells (Fig 2B and 2C). The inhibitory effects of BFA1 and LC were confirmed by inhibition of intracellular lysosomal degradation of FITC-conjugated dextran (for BFA1), and inhibition of proteasomal degradations of IκBα and cyclin B, and induction of caspase-3 (for LC) (S2A and S2B Fig). This result indicates that TSG101 overexpression also downregulates Gag via the lysosomal degradation pathway. In addition, co-localization of overexpressed TSG101 with Lysotracker and accumulation of Gag in endosomal/lysosomal-enriched fraction by TSG101 overexpression were previously reported [28, 29]. We thus further confirmed the localization of Gag and TSG101 with a lysosomal marker (LAMP1) and endosomal markers (early endosomes, EEA1; late endosomes, Rab7; and recycling endosomes; Rab11). HeLa cells transfected with pCAGGS/Gag and pCAGGS/TSG101 showed increase of Gag co-localization in lysosomes and endosomes (Fig 2D and S3 Fig).

Taken together, these results suggest that Vpr abrogates TSG101 overexpression effect to prevent Gag accumulation and degradation via endosomal/lysosomal pathway thereby rescuing VLP production.

### Vpr/Gag interaction is required to rescue VLP production affected by TSG101 overexpression

**Fig 3:**
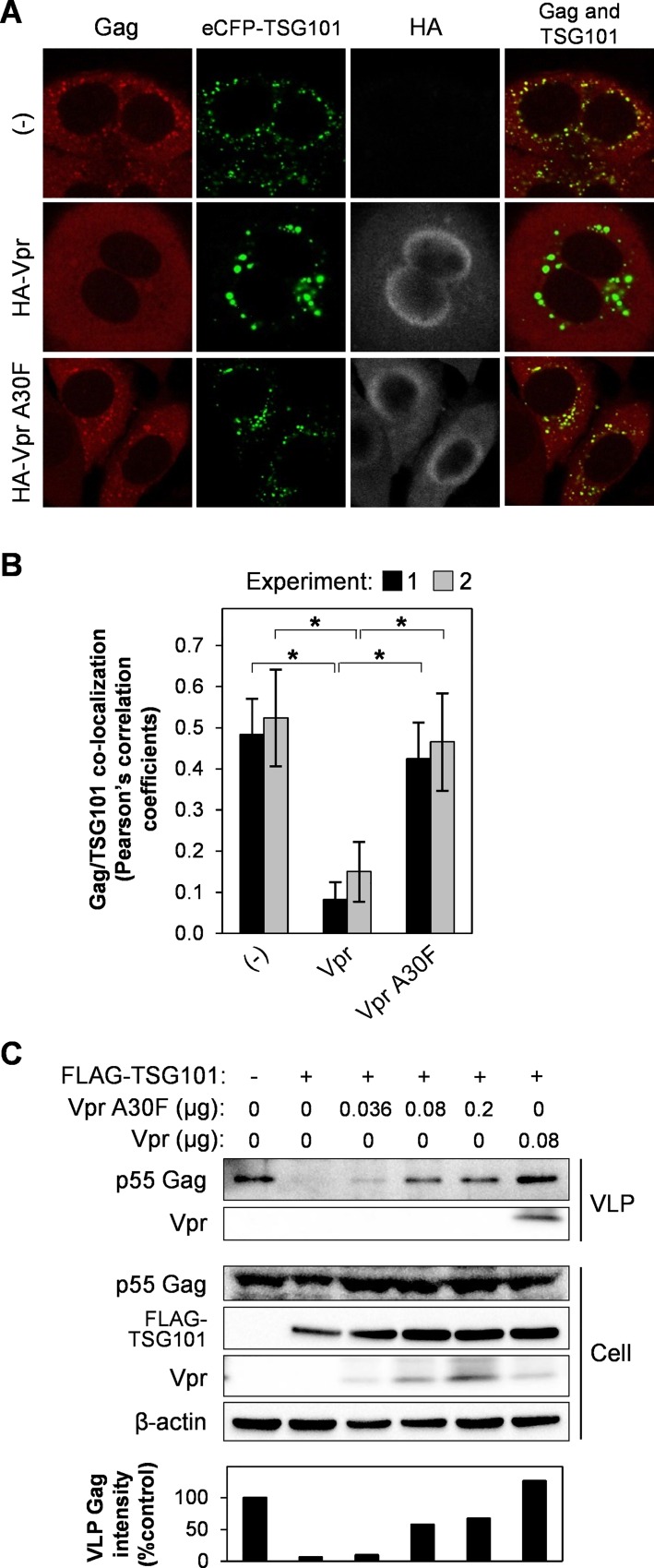
Vpr and Gag interaction is required to abrogate the effect of TSG101 overexpression. (A) HeLa cells were co-transfected with 0.8 μg of pCAGGS/Gag and 1.5 μg of pCAGGS/eCFP-TSG101 either without/with 0.5 μg of pCAGGS/HA-Vpr or pCAGGS/HA-Vpr A30F for 48 h prior to immunofluorescence staining with an anti-Gag and anti-HA antibodies, followed by an Alexa Fluor 594 goat anti-rabbit antibody and an Alexa Fluor 633 goat anti-mouse antibody. Co-localization of Gag/TSG101 was analyzed by Pearson’s correlation coefficients (B). Data represent the means ± SD of the result of two independent experiments. *, P < 0.05 (unpaired t-test). (C) HEK293T cells were transfected with 0.8 μg of pCAGGS/Gag, or with 0.8 μg of pCAGGS/Gag plus 1.5 μg of pCAGGS/FLAG-TSG101 either without/with different amounts of pCAGGS/Vpr A30F or 0.08 μg of pCAGGS/Vpr (positive control). After 48 h, VLPs in the cultured medium were collected by a 20% sucrose cushion. Whole cell lysates were prepared and samples were subjected to western blot analysis with anti-Gag, anti-Vpr, anti-FLAG, and anti-β-actin antibodies. The bottom panel represents intensity of VLP Gag from western blot analysis. Data represent the result of one representative experiment from two independent performs.

To verify whether Vpr/Gag interaction directly involves with the rescue effect of Vpr on VLP production, the Vpr A30F mutant which is unable to bind Gag and incorporate into viral particles was used [16, 38]. Co-transfection of pCAGGS/Gag and pCAGGS/eCFP-TSG101 with pCAGGS/HA-Vpr A30F did not inhibit Gag/TSG101 co-accumulation when compared with the wild-type Vpr (Fig 3A and 3B). This result correlates with the western blot analysis of VLP production showing partial rescue effect of Vpr A30F mutant against TSG101 overexpression (Fig 3C). No Vpr incorporation into VLP was detected in the presence of Vpr A30F mutant (Fig 3C), which confirms that this mutant is defective in Gag-mediated Vpr incorporation into virion [16, 38]. All together indicates that Vpr/Gag interaction is required to abrogate the TSG101 overexpression effects.

### Vpr rescues viral release from the TSG101 overexpression

**Fig 4:**
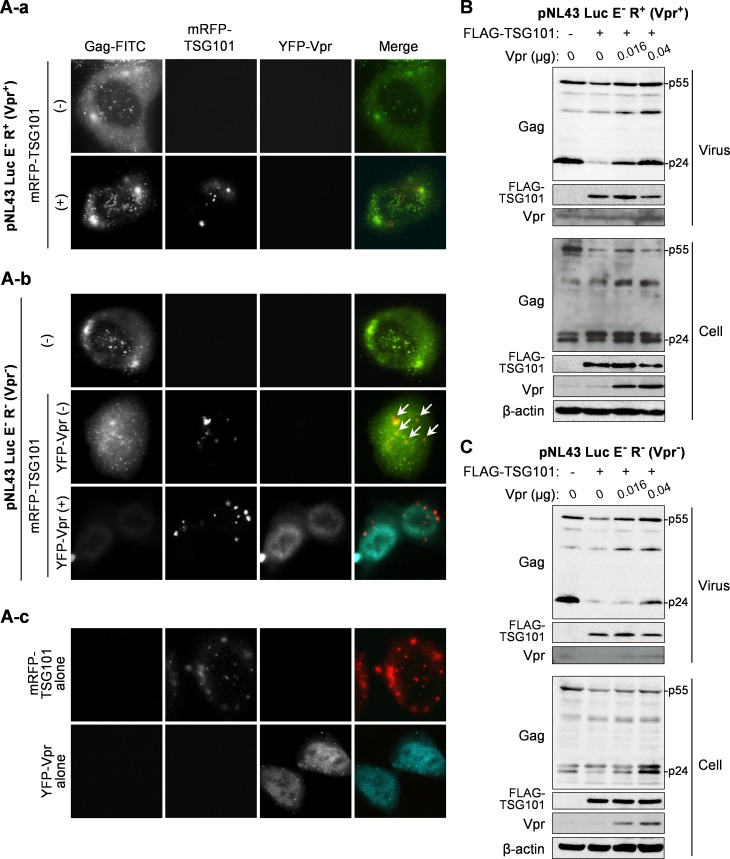
Vpr rescues the TSG101-mediated inhibition of viral particle release. (A) HeLa cells were transfected with 0.3 μg of pNL43 Luc E^-^ R^+^ (Vpr^+^) or pNL43 Luc E^-^ R^-^ (Vpr^-^) either without/with 0.1 μg of pCAGGS/mRFP-TSG101 and 0.1 μg of pcDNA/YFP-Vpr for 16 h prior to immunofluorescence staining with an anti-Gag AG3.0 antibody followed by a FITC-conjugated sheep anti-mouse antibody. White arrows indicate Gag/TSG101 co-localized on/near the plasma membrane observed by total internal reflection fluorescence (TIRF) microscopy. Data represents the result of one representative experiment from two independent performs. (B-C) HEK293T cells were transfected with 0.2 μg of pNL43 Luc E^-^ R^+^ (Vpr^+^) (B) or pNL43 Luc E^-^ R^-^ (Vpr^-^) (C), together with 1 μg of pCAGGS/FLAG-TSG101 and different amounts of pCAGGS/Vpr for 48 h. Viral particles in the culture medium were collected by PEG precipitation. Whole cell lysates were prepared and samples were subjected to western blotting with anti-Gag, anti-Vpr, anti-FLAG, and anti-β-actin antibodies. Data represents the result of one representative experiment from two independent performs.

We further confirmed the ability of Vpr to inhibit Gag/TSG101 co-accumulation by using an HIV-1 reporter plasmid that expresses or does not express intact Vpr protein (pNL43 Luc E^-^ R^+^ or pNL43 Luc E^-^ R^-^, respectively) in the presence/absence of TSG101 and Vpr expression plasmids. TIRF microscopy was used to show co-localization of Gag/TSG101 on the cell surface and at the intracellular region close to the plasma membrane. In addition to the perinuclear localization of overexpressed TSG101 (Figs 1 and 3), TIRF imaging indicated that TSG101 localized in a punctate pattern close to the plasma membrane (Fig 4A–4C). The viral Gag protein also localized in a punctate pattern (Fig 4A-a and 4A-b), which is a typical pattern associated with viral assembly and budding sites [39]. Co-transfection of pNL43 Luc E^-^ R^+^ (Vpr^+^) and pCAGGS/mRFP-TSG101 resulted in no co-localization of Gag/TSG101 (Fig 4A-a). However, the Gag/TSG101 co-accumulation was observed in cells transfected with pNL43 Luc E^-^ R^-^ (Vpr^-^) and pCAGGS/mRFP-TSG101 (Fig 4A-b). In addition, co-transfection of pcDNA/YFP-Vpr together with pNL43 Luc E^-^ R^-^ (Vpr^-^) and pCAGGS/mRFP-TSG101 abolished the effect of TSG101 overexpression on Gag accumulation (Fig 4A-b). Moreover, the lower Gag fluorescent signal observed on/near the plasma membrane in this sample may indicate efficient Vpr-induced virion release under the condition of TSG101 overexpression (Fig 4A-b).

Consistent with the result of the VLP production experiments, Vpr also counteracted the effects of TSG101 overexpression on viral particle production. Overexpression of TSG101 reduced intracellular p55 Gag expression and the levels of virion-derived p55 and p24 Gag in the culture medium from both pNL43 Luc E^-^ R^+^ (Vpr^+^) and pNL43 Luc E^-^ R^-^ (Vpr^-^) (Fig 4B and 4C). However, the effect of TSG101 overexpression was more obvious in the presence of the pNL43 Luc E^-^ R^-^ (Vpr^-^) plasmid (Fig 4C). The TSG101 effect on pNL43 Luc E^-^ R^+^ (Vpr^+^) might be due to high expression of the TSG101 plasmid thereby overcoming the effect of Vpr expressed from the pNL43 Luc E^-^ R^+^ plasmid (Fig 4B). As expected, co-transfection of pCAGGS/Vpr restored intracellular p55 Gag expression and virion production, and this effect was dependent on the amount of pCAGGS/Vpr plasmid (Fig 4B and 4C). The ability of Vpr to rescue virus production was lower for pNL43 Luc E^-^ R^-^ than for pNL43 Luc E^-^ R^+^ because the latter expresses intact Vpr (Fig 4B and 4C).

Taken together, these results verify a role of Vpr to overcome TSG101 overexpression effects on Gag accumulation, Gag degradation, and the defect in VLP/virion production.

### Vpr competes with TSG101 to bind Gag

**Fig 5:**
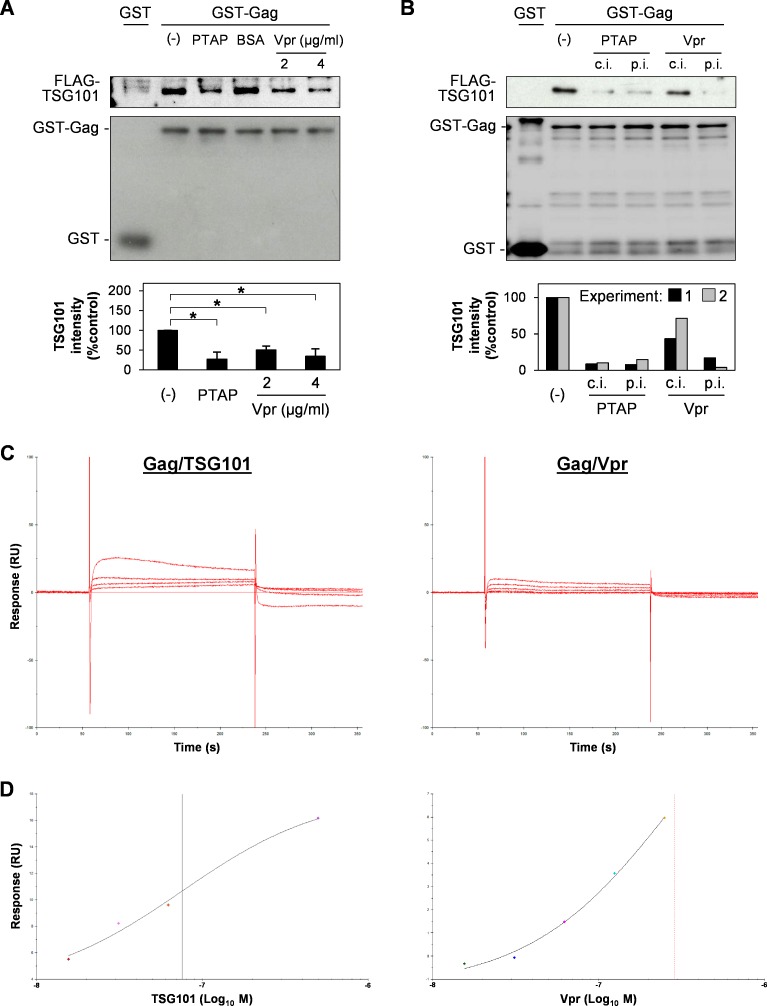
Vpr competes with TSG101 for binding to Gag. (A) GST or GST-Gag protein were immobilized on Glutathione Sepharose beads prior to overnight incubation with purified FLAG-TSG101 protein in the absence/presence of 48.3 μg/ml of PTAP short peptide (a positive control which targets the UEV domain of TSG101), 48.3 μg/ml of BSA (negative control), or 2.0 or 4.0 μg/ml of purified HA-Vpr protein. The beads were pulled-down, washed, and subjected to western blotting with anti-FLAG and anti-GST antibodies. The bottom panel represents mean ± SD intensity values of Gag-bound TSG101 from western blot analysis of four independent performs. *, P < 0.05 (unpaired t-test). (B) GST-Gag was co-incubated with purified FLAG-TSG101 and 48.3 μg/ml of PTAP short peptide or 2 μg/ml of HA-Vpr protein (competitive incubation, c.i.), or allowed to bind with PTAP short peptide or HA-Vpr protein for 3 h before co-incubation with FLAG-TSG101 protein (pre-incubation, p.i.). After overnight incubation, the beads were pulled-down, washed, and subjected to western blotting with anti-FLAG and anti-GST antibodies. The bottom panel represents intensity of Gag-bound TSG101 from western blot analysis of two independent performs. (C-D) GST-Gag protein was immobilized on Biacore sensor chip CM5 and the surface was injected with purified FLAG-TSG101 or FLAG-Vpr protein at different concentrations by Biacore T100 instrument. Kinetic sensorgrams of Gag-bound TSG101 at 0, 15.6, 31.3, 62.5, and 500.0 nM (C, left panel) and Gag-bound Vpr at 0, 15.6, 31.3, 62.5, 125.0, and 250.0 nM (C, right panel) are showed. (D) Steady stage affinity fitting curves of Gag/TSG101 (left panel) and Gag/Vpr (right panel) binding at equilibrium (4 s before injection stop). Black and red vertical lines represent equilibrium dissociation constant (KD) of Gag/TSG101 and Gag/Vpr binding affinity, representatively. Data represents the result of one representative experiment from two independent performs.

To investigate the mechanism underlying Vpr-mediated inhibition of the effects of TSG101 overexpression, we conducted a competitive pull-down assay using Gag, TSG101, and Vpr. GST or GST-Gag was immobilized on Glutathione Sepharose beads and co-incubated with FLAG-TSG101 protein in the absence or presence of HA-Vpr protein. A PTAP short peptide inhibitor targeting the UEV domain of TSG101 [35] was used as a positive control to inhibit Gag/TSG101 binding while BSA was used as a negative control. The results showed that Gag/TSG101 binding was reduced in the presence of PTAP or Vpr which indicates that Vpr competes with TSG101 for Gag (Fig 5A). In addition, allowing Vpr to bind Gag for 3 h prior to addition of the TSG101 protein reduced the Gag/TSG101 binding even further (Fig 5B). There was no difference in the effect of PTAP under pre-incubation condition, since the PTAP short peptide targets the TSG101 protein. Binding kinetic and affinity of Gag/TSG101 and Gag/Vpr were then compared by Biacore analysis using Gag immobilized sensor chip surface. The surface was injected with different concentrations of purified FLAG-TSG101 or FLAG-Vpr protein for response measurement. Response data from two independent experiments was then individually fitted into 1:1 Binding model for kinetic analysis or Steady Stage Affinity model for affinity analysis. Sensorgrams of Gag/TSG101 and Gag/Vpr binding kinetic suggest rapid association and dissociation of both interactions (Fig 5C). TSG101 faster associated and dissociated with Gag at the association rate constant (k_a_) of 9.24 × 10^5^ M^-1^s^-1^ and dissociation rate constant (k_d_) of 2.23 × 10^−2^ s^-1^ when compared with Vpr (k_a_ = 3.73 × 10^5^ M^-1^s^-1^ and k_d_ = 7.19 × 10^−3^ s^-1^). In addition, the binding affinity of Gag/TSG101 (KD = 55.18 nM) was only 6.2-fold higher than that of Gag/Vpr (KD = 342.58 nM) (Fig 5D). These results may support the possibility of Vpr to compete TSG101 for Gag interaction.

All together, these data suggest that Vpr competes with TSG101 for Gag binding, thereby inhibiting Gag/TSG101 co-accumulation and Gag degradation, and rescuing defective VLP/virion production.

## Discussion

Here, we identified a molecular mechanism by which Vpr overcomes the effect of TSG101 overexpression to rescue viral production. Co-expression of Vpr prevents Gag accumulation in endosomal compartments, inhibits Gag degradation in lysosome, and rescues viral production under TSG101 overexpression condition. Although, Vpr mainly localizes in the nucleus [40], some molecules are present in the perinuclear region [41] and cytoplasm, where they may counteract the effects of TSG101 overexpression. This inhibitory effect of Vpr may be due to interference of Gag/TSG101 binding, since the Gag/Vpr binding site (^15^FRFG^18^) [19] and Gag/TSG101 (^7^PTAP^10^) binding site are close to each other; indeed, our GST pull-down assays revealed the competition between Vpr and TSG101 for Gag binding. In addition, Biacore analysis shows 6.2-fold lower binding affinity and 3.1-fold slower dissociation kinetic of Gag/Vpr when compare with Gag/TSG101 which indicates the possibility of Vpr to compete TSG101 for Gag. The competitive binding effect of Vpr *in vitro* (GST pull-down) is lower than that of *in vivo* (immunofluorescences and VLP/virus production) which may result from the difference of binding conditions between *in vitro* and *in vivo*. Vpr may block the TSG101-mediated accumulation of Gag in perinuclear region. Gag is then trafficked to the plasma membrane and viral assembly/budding occurs. This TSG101 counteracting effect of Vpr did not occur as efficiently when using Vpr A30F mutant which is defective in Gag binding and Gag-mediated Vpr virion incorporation [16, 38]. In addition, TSG101 overexpression did not cause accumulation of Gag LIRL, a PTAP domain mutant which does not interact with TSG101. This suggests that the Gag/Vpr interaction is required to rescue the effects of TSG101 overexpression.

Intracellular Pr55 Gag is post-translationally modified by the addition of a myristoyl group at the N-terminus; this targets Gag to the plasma membrane where viral assembly/budding takes place [1]. Gag must interact with TSG101 to initiate virion assembly/budding via the ESCRT pathway. The results presented herein and previous publications [28, 29] show that overexpression of TSG101 causes the protein to localize at the early/late endosomes and lysosomes. The functions of TSG101 on MVB biogenesis [6, 7] may explain this localization pattern; however, additional experiments are required to support this. Co-expression of TSG101 and Gag shifts Gag localization from the plasma membrane/cytoplasm to the endosomes and lysosomes. This may initially occur via recruitment of plasma membrane/cytoplasm-localized Gag by the early endosome-localized TSG101; Gag may then be internalized into the MVBs in the lumen of early endosomes by TSG101, followed by transportation to late endosomes. The MVB-Gag thus mainly accumulates in the endosomal compartment, which subsequently fuses with lysosomes, resulting in Gag degradation. This pathway may be similar to that used by TSG101/Hrs interaction to regulate expression of the cell surface epidermal growth factor receptor (EGFR) molecules [42]. Hrs is an endosomal protein that interacts with TSG101 via its PSAP motif in a manner similar to the Gag-PTAP/TSG101 interaction [43]. This TSG101/Hrs interaction sequentially induces the formation of EGFR-MVBs in the endosomal lumen, which are then trafficked to lysosomes for degradation [42]. In addition, expression of Gag modulates TSG101-mediated downregulation of EGFR, whereas expression of C-terminal Hrs interferes with TSG101-mediated HIV-1 Gag particle release [44, 45]. These two studies indicate that TSG101 regulates the intracellular and extracellular formation of membrane particles. However, it is unclear whether Hrs participates in Gag accumulation in endosomes under conditions of TSG101 overexpression.

Harila *et al*. [46] showed that Vpu regulates Pr55 Gag targeting to the plasma membrane for extracellular viral particle budding, or to the internal membrane for intracellular viral particle budding. A newly synthesized Pr55 Gag is initially targeted to plasma membrane, where it remains in the presence of viral Vpu for cell surface viral budding. Without Vpu, Pr55 Gag relocates from the plasma membrane to internal membrane compartments (endosomes and lysosomes) via the endocytosis pathway for intracellular viral budding [46]. In addition, re-localization of Pr55 Gag is dependent on the presence of TSG101 or the Gag p6 domain [46]. These data support our results showing that TSG101 overexpression induces Gag re-localization to endosomal compartments. However, we found that further Gag degradation occurs via the endosomal/lysosomal pathway. It will be interesting to examine whether TSG101 overexpression promotes virus budding in internal compartments. In addition, comparing the abilities of Vpr and Vpu to rescue the effects of TSG101 overexpression may provide useful information that will improve our understanding of the role played by viral accessory proteins in the assembly/budding process.

## Supporting Information

S1 Fig TSG101 overexpression induces Gag accumulation via the Gag PTAP domain and Vpr prevents this accumulation. (TIF)

S2 Fig Control experiments for determination of Bafilomycin A1 and Clasto-lactacystin β-lactone effects. (TIF)

S3 Fig TSG101 overexpression induces Gag accumulation in endosomes and lysosomes. (TIF)

S1 File Supporting Material and Methods and Supporting Figure Legends. (DOCX)
