# Supplementary figures and images for: HIV-1 Vpr Abrogates the Effect of TSG101 Overexpression to Support Virus Release
Source: PLoS One. 2016 Sep 20;11(9):e0163100. doi: 10.1371/journal.pone.0163100 (PMC5029901; doi:10.1371/journal.pone.0163100)

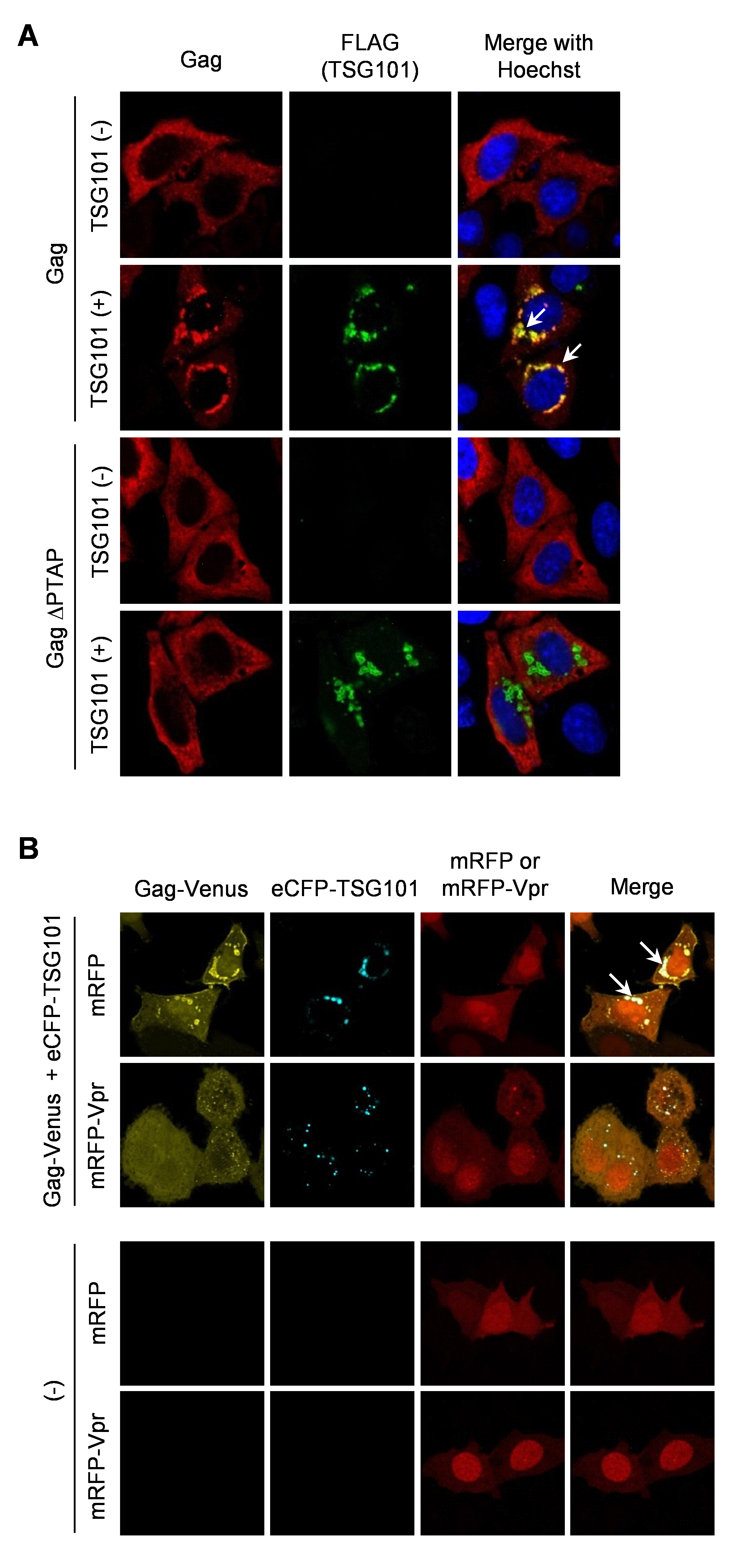

Supplement: S1 Fig — (TIF) [file pone.0163100.s001.tif]

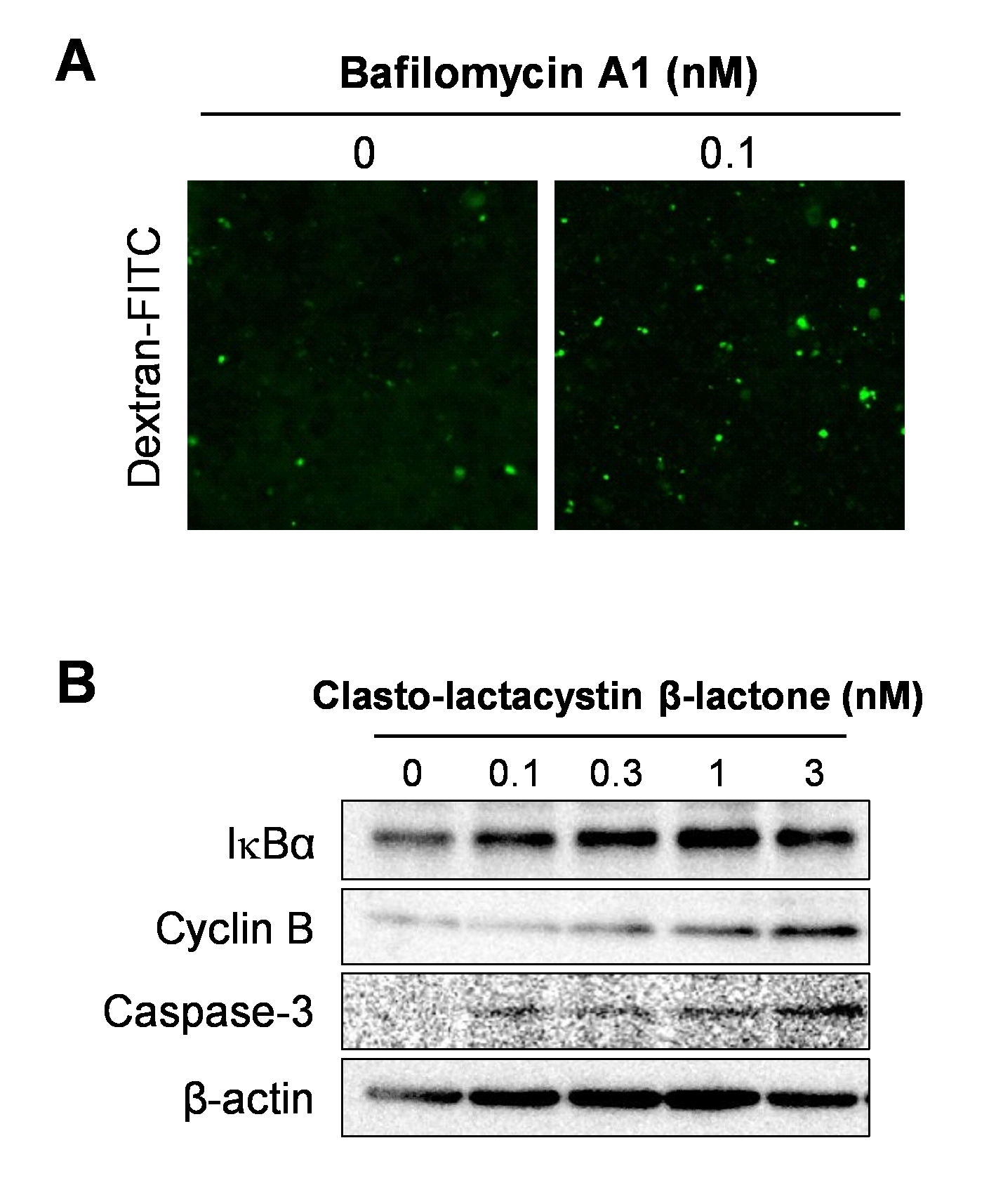

Supplement: S2 Fig — (TIF) [file pone.0163100.s002.tif]

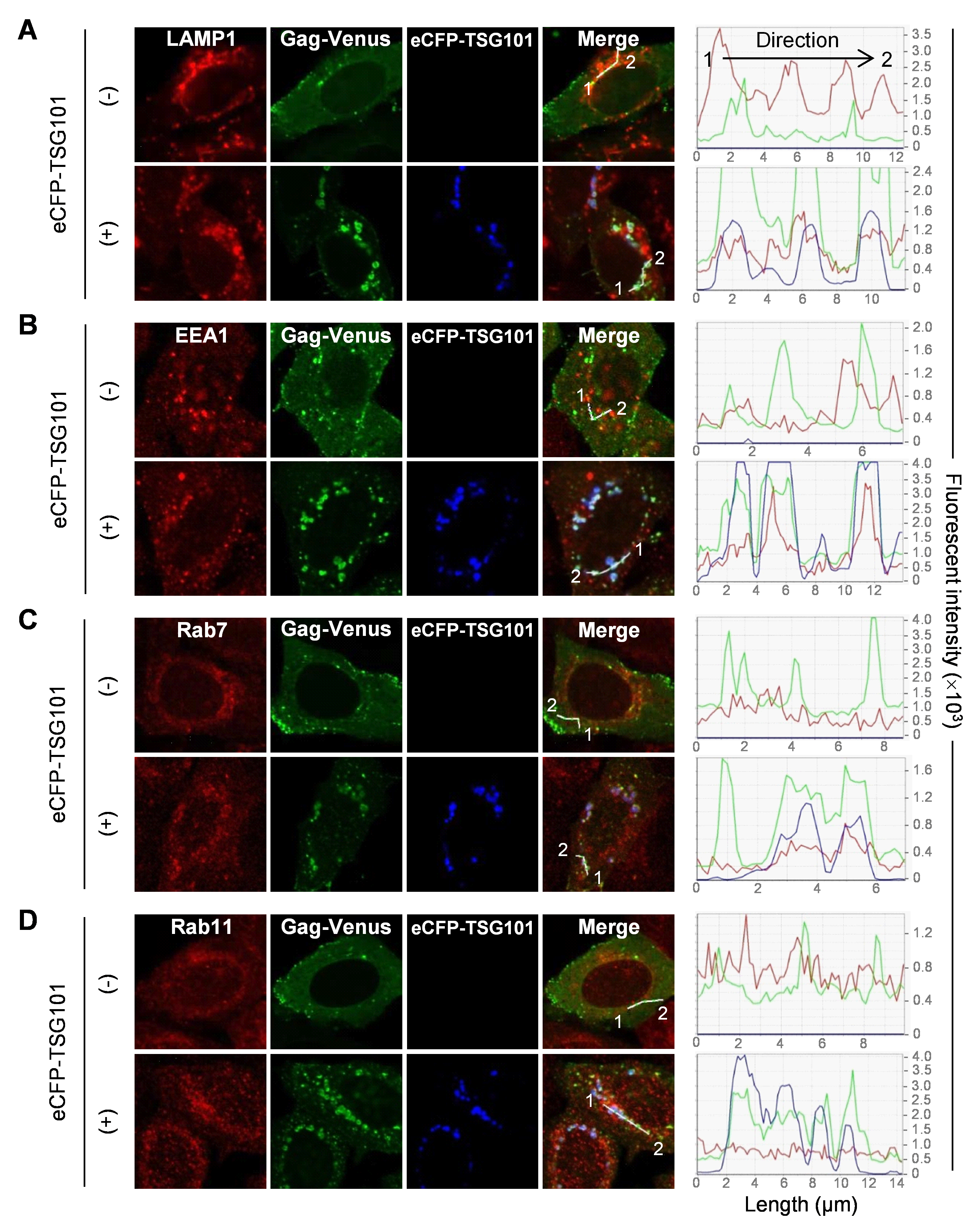

Supplement: S3 Fig — (TIF) [file pone.0163100.s003.tif]
