# Supplementary material for: HIV-1 Vpr Abrogates the Effect of TSG101 Overexpression to Support Virus Release
Source: PLoS One. 2016 Sep 20;11(9):e0163100. doi: 10.1371/journal.pone.0163100 (PMC5029901; doi:10.1371/journal.pone.0163100)
Supplement: S1 File — (DOCX) [file pone.0163100.s004.docx]

**Supporting Information**

**Supporting Material and Methods**

Plasmid construction

The pNL432-derived pNF462 HIV-1 molecular clone plasmid (a gift from Dr. Akio Adachi, Tokushima University, Japan) [1] was used as a template for Gag plasmid construction. pGEX-6P-3/Gag was constructed by amplifying the Gag sequence using KOD Plus DNA polymerase (Toyobo) with forward primer, 5'-TAGGATCCAGCGGTGCGAGAGCGTCGG-3', and reverse primer, 5'-GCCTCGAGTTATTGTGACGAGGGGTCGC-3', followed by sub-cloning into the *BamHI/XhoI* sites of pGEX-6P-3 (GE Healthcare Life Sciences). pCAGGS/Gag was constructed using forward primer, 5'-AAAACTCGAGATGGGTGCGAGAGCGTCGGTA-3' and reverse primer, 5'-AAAAGCGGCCGCTTATTATTATTGTGACGAGGGGTC-3', followed by sub-cloning into the *XhoI/NotI* sites of the modified pCAGGS plasmid [2]. To construct pCAGGS/Venus and pCAGGS/eCFP, the Venus sequence was amplified by forward primer, 5'- AAACTCGAGCCGCCACCATGGGTACCGCAGCGGCTGCAGCGGCTGTGAGCAAGGGCGAGGAGCTGTTCA-3' and reverse primer, 5'-TTTGCGGCCGCTTATCTATTCACTTGTACAGCTCGTCCATGCCGAGA-3', and the eCFP sequence was amplified by forward primer, 5'-AAACTCGAGCCGCCACCATGGTGAGCAAGGGCGAGGAGCTGTT-3' and reverse primer, 5'-TTTGCGGCCGCTTATCTATTCACCCGGGAGCCGCTGCAGCCGCTGCCTTGTACAGCTCGTCCATGCCGAGA-3', from pCS2/Venus and pCS2/eCFP, respectively (a gift from Dr. Atsushi Miyawaki, RIKEN Brain Science Institute), followed by sub-cloning into the *XhoI/NotI* sites of the modified pCAGGS vector. pCAGGS/Gag-Venus was constructed by amplifying the Gag sequence with forward primer, 5'-AACTCGAGACCATGGGTGCGAGAGCGTCGGTA-3' and reverse primer, 5'-AAGGTACCTTGTGACGAGGGGTCGCT-3', followed by sub-cloning into the *XhoI/KpnI* sites of pCAGGS/Venus.

pCAGGS/Vpr and pCAGGS/HA-Vpr were constructed from pME18Neo/FLAG-Vpr plasmid [3, 4] using forward primer (for Vpr), 5'-AAAACTCGAGATGGAACAAGCCCCAGAAGAC-3' or forward primer (for HA-Vpr), 5'-ACTCGAGATGTACCCATACGATGTTCCAGATTACGCTGAACAAGCCCCAGAAGAC-3', with reverse primer, 5'-AAAAGCGGCCGCTTATCACTAGGATCTACTGGCTCC-3', followed by sub-cloning into the *XhoI/NotI* sites of the modified pCAGGS. pCAGGS/Vpr A30F mutant was constructed by site direct mutagenesis using PrimeSTAR Max (Takara) with forward primer, 5’-AGTGAATTTGTTAGACATTTTCCTAG-3’ and reverse primer, 5'-CTAACAAATTCACTCTTAAGTTCCTC-3'. pCAGGS/mRFP-Vpr was constructed using two primer sets: forward primer, 5'-AACTCGAGACCATGGCCTCCTCCGAGGACGTC-3' and reverse primer, 5'-AACCCGGGGCTGCCGGCGCCGGTGGAGTGGCG-3' for mRFP sequence amplification, and forward primer, 5'-AACCCGGGAGCGGCGAACAAGCCCCAGAAGAC-3' and reverse primer, 5'-AAGCGGCCGCTTACTAGGATCTACTGGCTCCA-3' for Vpr sequence amplification. These fragments were then digested with *XhoI/XmaI* or *XmaI/NotI*, respectively, and sub-cloned into the *XhoI/NotI* sites of the modified pCAGGS vector. pcDNA/YFP-Vpr was constructed by amplifying the YFP sequence with forward primer, 5'-GGGCTAGCACCATGGTGAGCAA-3' and reverse primer, 5'-GGCTCGAGCTTGTACAGCTCGTCCATGCCGAGAG-3' (from pYFP-C1; BD Biosciences Clontech), and the Vpr sequence with forward primer, 5'-GGGCTCGAGGAACAAGCCCCAGAAGA-3' and reverse primer, 5'-GGGGCGGCCGCTCGAGCTAGGATCT-3' (from pNL43). Both fragments were then sub-cloned into the *NheI/NotI* sites of pcDNA 3.1 (Invitrogen).

Total cDNAs derived from HeLa cells was used as templates to construct the TSG101 plasmids. pCAGGS/FLAG-TSG101 was constructed using forward primer, 5'-TACTCGAGATGGATTACAAGGATGACGACGATAAGGCGGTGTCGGAGAGCCAG-3' and reverse primer, 5'-GAGCGGCCGCTCAGTAGAGGTCACTGAGACCGG-3', followed by sub-cloning into the *Xho/NotI* sites of the modified pCAGGS vector. pCAGGS/eCFP-TSG101 and pCAGGS/mRFP-TSG101 were construct by amplifying the TSG101 sequence with forward primer, 5'-AAACCCGGGGCGGTGTCGGAGAGCCAG-3' and reverse primer, 5'-AAAGCGGCCGCTCATCAGTAGAGGTCACTGAG-3', followed by sub-cloning into the *XmaI/NotI* sites of pCAGGS/eCFP or pCAGGS/mRFP-Vpr (replaced Vpr sequence with TSG101).

HIV-1 *env^-^* reporter plasmids, pNL43 Luc E^-^ R^+^ (Vpr^+^) and pNL43 Luc E^-^ R^-^ (Vpr^-^), were provided by Dr. Kenzo Tokunaga (National Institute of Infectious Diseases, Tokyo, Japan) [5].

All constructed plasmids were checked by DNA sequencing using a BigDye Terminator v.3.1 Cycle Sequencing Kit (Applied Biosystems).

**Supporting References**

1. Kawamura, M., Ishizaki, T., Ishimoto, A., Shioda, T., Kitamura, T., and Adachi, A. (1994). Growth ability of human immunodeficiency virus type 1 auxiliary gene mutants in primary blood macrophage cultures. J. Gen. Virol. *75*, 2427-2431.
2. Hagiwara, K., Murakami, T., Xue, G., Shimizu, Y., Takeda, E., Hashimoto, Y., Honda, K., Kondoh, Y., Osada, H., Tsunetsugu-Yokota, Y.*, et al.* (2010). Identification of a novel Vpr-binding compound that inhibits HIV-1 multiplication in macrophages by chemical array. Biochem. Biophys. Res. Commun. *403*, 40-45.
3. Nishino, Y., Myojin, T., Kamata, M., and Aida, Y. (1997). Human immunodeficiency virus type 1 Vpr gene product prevents cell proliferation on mouse NIH3T3 cells without the G2 arrest of the cell cycle. Biochem. Biophys. Res. Commun. *232*, 550-554.
4. Nishizawa, M., Myojin, T., Nishino, Y., Nakai, Y., Kamata, M., and Aida, Y. (1999). A carboxy-terminally truncated form of the Vpr protein of human immunodeficiency virus type 1 retards cell proliferation independently of G(2) arrest of the cell cycle. Virology *263*, 313-322.
5. Adachi, A., Gendelman, H.E., Koenig, S., Folks, T., Willey, R., Rabson, A., and Martin, M.A. (1986). Production of acquired immunodeficiency syndrome-associated retrovirus in human and nonhuman cells transfected with an infectious molecular clone. J. Virol. *59*, 284-291.
6. Yoshimori T, Yamamoto A, Moriyama Y, Futai M, Tashiro Y. Bafilomycin A1, a specific inhibitor of vacuolar-type H(+)-ATPase, inhibits acidification and protein degradation in lysosomes of cultured cells. J Biol Chem. 1991;266(26):17707-12. Epub 1991/09/15. PubMed PMID: 1832676.
7. Lin KI, Baraban JM, Ratan RR. Inhibition versus induction of apoptosis by proteasome inhibitors depends on concentration. Cell Death Differ. 1998;5(7):577-83. Epub 1999/04/14. doi: 10.1038/sj.cdd.4400384. PubMed PMID: 10200512.
8. Choi YH, Lee SJ, Nguyen P, Jang JS, Lee J, Wu ML, et al. Regulation of cyclin D1 by calpain protease. J Biol Chem. 1997;272(45):28479-84. Epub 1997/11/14. PubMed PMID: 9353308.
9. Chen L, Smith L, Wang Z, Smith JB. Preservation of caspase-3 subunits from degradation contributes to apoptosis evoked by lactacystin: any single lysine or lysine pair of the small subunit is sufficient for ubiquitination. Mol Pharmacol. 2003;64(2):334-45. Epub 2003/07/19. doi: 10.1124/mol.64.2.334. PubMed PMID: 12869638.

**Supporting Figure Legends**

**S1 Fig. TSG101 overexpression induces Gag accumulation via the Gag PTAP domain and Vpr prevents this accumulation.** (A) HeLa cells were transfected with 0.8 µg of pCAGGS/Gag or pCAGGS/Gag ∆PTAP (LIRL) either without/with 1.5 µg of pCAGGS/FLAG-TSG101 for 48 h followed by immunofluorescence staining with an anti-Gag and anti-FLAG antibodies together with an Alexa Fluor 594 goat anti-rabbit antibody and an Alexa Fluor 488 goat anti-mouse antibody. Nuclei were stained with Hoechst 33342. White arrows indicate Gag and TSG101 co-localization. (B) HeLa cells were transfected with 0.8 µg of pCAGGS/Gag-Venus and 1.5 µg of pCAGGS/eCFP-TSG101 either without/with 0.5 µg of pCAGGS/mRFP or 0.5 µg of pCAGGS/mRFP-Vpr, or with 0.5 µg of pCAGGS/mRFP or 0.5 µg of pCAGGS/mRFP-Vpr only for 48 h followed by fixation before visualization. White arrows indicate Gag and TSG101 co-localization.

**S2 Fig. Control experiments for determination of Bafilomycin A1 and Clasto-lactacystin β-lactone effects.**

(A) HEK293T cells were seeded overnight and incubated with 0.5 mg/ml of FITC-conjugated dextran for 24 h in CO_2_ incubator. The culture medium was replaced and the cells were treated with Bafilomycin A1 for 6 h before image acquisition at Ex./Em. = 488/519 nm for observation of lysosomal degradation inhibition [6]. (B) HEK293T cells were seeded overnight and treated with different concentrations of Clasto-lactacystin β-lactone for 6 h. The cells were collected and lysed for western blotting with markers for proteasomal inhibition, i.e., IκBα [7], cyclin B [8], and caspase-3 [9].

**S3 Fig.** **TSG101 overexpression induces Gag accumulation in endosomes and lysosomes.** (A-D) HeLa cells were transfected with 0.8 µg of pCAGGS/Gag-Venus, or with 0.8 µg of pCAGGS/Gag-Venus plus 1.5 µg of pCAGGS/eCFP-TSG101, for 48 h, followed by immunofluorescence staining with the following antibodies against lysosomal or endosomal markers: LAMP1 (A), EEA1 (B), Rab7 (C), and Rab11 (D). The secondary antibody was Alexa Fluor 594 goat anti-rabbit antibody. The following color changes were made to facilitate visualization of co-localization: Venus to green and eCFP to blue. (A-D, right) Fluorescent intensity plot of lysosomal or endosomal markers (red), Gag (green), and TSG101 (blue) derived from the white line in the merge channel (from the direction 1 to 2).
